# Characteristics of chest pain in COVID-19 patients in the emergency department

**DOI:** 10.1007/s12471-022-01730-7

**Published:** 2022-10-21

**Authors:** M. Sinkeldam, A. G. Buenen, E. Celiker, M. van Diepen, A. M. de Vos

**Affiliations:** 1 Department of Intensive Care, Canisius-Wilhelmina Hospital, Nijmegen, The Netherlands; 2 Department of Emergency Medicine, Bernhoven Hospital, Uden, The Netherlands; 3 Department of Cardiology, Canisius-Wilhelmina Hospital, Nijmegen, The Netherlands; 4 Department of Emergency Medicine, Maxima Medical Centre, Veldhoven, The Netherlands; 5 Radboud University, Nijmegen, The Netherlands; 6 Department of Cardiology, Bernhoven Hospital, Uden, The Netherlands

**Keywords:** COVID-19, Chest pain, Syncope, Arrhythmia

## Abstract

**Introduction:**

Patients with coronavirus disease 2019 (COVID-19) can present with chest pain. However, the characteristics of this chest pain are unknown. We performed a single-centre observational study to review and summarise chest pain characteristics in COVID-19 patients at first presentation to the emergency department (ED).

**Methods:**

We collected data on characteristics of ‘chest pain’ reported by COVID-19 patients who attended the ED of Bernhoven Hospital, the Netherlands from 4 through 30 March 2020.

**Results:**

We included 497 COVID-19 patients, of whom 83 (17%) reported chest pain upon presentation to the ED. Chest pain characteristics were: present since disease onset (88%), retrosternal location (43%), experienced as compressing/pressure pain (61%), no radiation (61%) and linked to heavy coughing (39%). Patients who reported chest pain were younger than those without chest pain (61 vs 73 years; *p* < 0.001). Patients with syncope were older (75 vs 72 years; *p* = 0.017), had a shorter duration of symptoms (5 vs 7 days; *p* < 0.001) and reported fewer respiratory complaints (68% vs 90%; *p* < 0.001) than those without syncope. Patients with new-onset atrial arrhythmias presented with a shorter duration of symptoms (5 vs 7 days; *p* = 0.013), experienced fewer respiratory complaints (72% vs 89%; *p* = 0.012) and more frequently had a history of cardiovascular disease (79% vs 50%; *p* = 0.003) than patients who presented without arrythmias.

**Conclusion:**

Chest pain and other cardiac symptoms were frequently observed in COVID-19 patients. Treating physicians should be aware that chest pain, arrhythmias and syncope can be presenting symptoms of COVID-19.

**Supplementary Information:**

The online version of this article (10.1007/s12471-022-01730-7) contains supplementary material, which is available to authorized users.

## What’s new?

- Patients with coronavirus disease 2019 (COVID-19) who presented to the emergency department (ED) with chest pain were younger than patients without chest pain.
- Typical COVID-19-related chest pain was mainly located retrosternally, had been present since the disease onset and was described as a compressing/pressure pain.
- Patients who presented with syncope due to COVID-19 were older, had a shorter duration of symptoms and had fewer respiratory complaints.
- Patients with new-onset atrial dysrhythmias and COVID-19 had a shorter duration of symptoms and fewer respiratory complaints than patients who presented without arrhythmias.
- In patients presenting to the ED with unexplained cardiac complaints, clinicians should be wary of COVID-19 as a potential cause.

## Introduction

Patients with coronavirus disease 2019 (COVID-19) typically present to the emergency department (ED) with signs and symptoms of respiratory tract infection, such as cough and dyspnoea combined with myalgia, malaise or headache [1–3]. However, one in four patients has cardiac manifestations, which they report as ‘chest pain’, ‘chest distress’ or ‘chest tightness’ [2–6]. Chest pain refers to discomfort or pain somewhere between the neck and the abdomen [7]. The underlying mechanism of chest pain in COVID-19 is unclear, but it may result from cardiac injury or pleural inflammatory infection [8]. Evidence of actual myocardial injury—defined as an elevated cardiac troponin level—is common among patients hospitalised with COVID-19 and is associated with a worse prognosis [7, 9, 10].

Other cardiac manifestations of COVID-19 upon presentation to the ED are syncope and arrhythmias. Syncope may be an isolated symptom in a COVID-19 patient [11–13]. It is an abrupt, transient and complete loss of consciousness associated with the inability to maintain postural tone, with rapid and spontaneous recovery [14]. Syncope is the presenting symptom of COVID-19 for 3–24% of patients presenting to the ED [11, 12, 15]. Furthermore, acute pulmonary embolism is a prognostically relevant complication of COVID-19, which can also present with syncope [16]. Additionally, co-infections and superinfections are common in COVID-19 patients [17]. The COVID-19 pandemic has resulted in a different approach to a patient presenting to the ED with syncope, because a missed or delayed diagnosis of COVID-19 due to an unusual presentation could lead to preventable exposures and increased transmission [15].

Arrhythmias are a potential cardiovascular complication of COVID-19 and occur in 9–28% of patients with COVID-19 and in up to 44% of those with severe COVID-19. Furthermore, they are associated with a worse prognosis [18–20]. In the COVID-19 population, arrhythmias develop secondary to hypoxaemia, metabolic dysregulation, electrolyte disorder, systemic inflammation, electrical instability with adrenergic stress, acute myocardial infarction or myocarditis, and treatment with QT-prolonging drugs [20].

We performed a single-centre observational study to assess the frequency and characteristics of chest pain in COVID-19 patients at first presentation to the ED. In addition, we investigated the occurrences of syncope and arrhythmias.

## Methods

### Patient selection and data collection

We collected data on baseline characteristics and symptoms of all COVID-19 patients attending the ED of Bernhoven Hospital, Uden, the Netherlands from 4 through 30 March 2020. For this purpose, a plug-in was added to the electronic health record (EHR) system, after which the attending physician had to check a box in the EHR to register and collect the data (f.e. check the box ‘hypertension in medical history’). In addition, all EHRs of the included COVID-19 patients were retrospectively assessed by at least two investigators (EC, MvD).

As Uden and its surroundings were the epicentre of the first wave of the COVID-19 pandemic in the Netherlands, general practitioners (GPs) were assigned a crucial role in treating patients at home to ensure the continuous patient flow remained manageable. Hence, only severe cases were referred to our ED. Additionally, numerous patients seen in the ED were admitted to nursing homes to provide adequate care.

This study was performed in line with the principles of the Declaration of Helsinki, and the institutional board approved this study. Collected data were stored and analysed anonymously. Patients were informed that their anonymised data could be used for research purposes, but formal approval from a medical ethics committee was not required as the Dutch Medical Research Involving Human Subjects Act (*Wet medisch-wetenschappelijk onderzoek met mensen*) did not apply to this observational study.

### COVID-19 diagnosis

A diagnosis of COVID-19 was made using an in-house real-time reverse transcriptase–polymerase chain reaction test targeting the *RdRp* gene of severe acute respiratory syndrome coronavirus 2 (SARS-CoV-2) on a deep naso-oropharyngeal swab. The indication for testing patients was presence of clinical features of COVID-19, such as fever, respiratory complaints and chest pain. Post-cardiac arrest patients and patients with unexplained diarrhoea were also tested.

### Symptom assessment

If present, symptoms such as fever, coughing, dyspnoea, respiratory complaints, diarrhoea, abdominal pain, and chest distress, chest pain or chest tightness were carefully recorded for each patient. The following comorbidities were documented: cardiovascular disease, pulmonary disease, chronic renal disease, hypertension, diabetes mellitus, active malignancy and obesity (body mass index ≥ 30 kg/m^2^).

To analyse the characteristics of the chest pain, the amount of information recorded in the EHRs needed to be sufficient. All patients who presented to the ED were asked whether they had any chest pain or chest complaints. If applicable, we recorded the chest pain characteristics such as the moment of first noticing them, characteristics of the pain, location of the pain, radiation to other parts of the body and provoking or relieving factors.

During data collection (in March 2020), the occurrence of thrombo-embolic events in COVID-19 patients was not as well-known and documented as it is nowadays. Hence, we did not routinely perform a D-dimer test in COVID-19 patients who presented with chest pain. Furthermore, cardiac markers, such as (high-sensitivity) troponin, were not routinely assessed because of the exceptionally high incidence of COVID-19.

### Statistical analysis

Continuous variables were analysed with a Mann-Whitney U test and discrete variables with a Fisher’s exact test. Data are reported as number (percentage) or median (range). For statistical analyses, we used IBM SPSS Statistics 26 (IBM Corp, Armonk, NY, USA). A *p*-value ≤ 0.05 was considered statistically significant.

## Results

### Baseline characteristics

A total of 497 patients with COVID-19 were included, of whom 317 (64%) were male (Tab. 1). Median age upon presentation was 72 years.

**Table 1:** Baseline characteristics

Variable	Patients (*N* = 497)
Male	317 (64)
Age, years	72 (27–94)
Symptom duration, days	7 (0–22)
*Comorbidities*	
Cardiovascular disease	256 (52)
Pulmonary disease	129 (26)
Renal disease	97 (20)
Hypertension	259 (52)
Diabetes mellitus	102 (21)
Active malignancy	35 (7)
Obesity (BMI ≥ 30 kg/m^2^)	151 (30)
*Symptoms*	
Fever	399 (80)
Respiratory complaints	435 (88)
Gastrointestinal complaints	261 (53)
Chest pain	83 (17)
Syncope	63 (13)
Arrythmias	
– None	382 (82)
– New-onset atrial arrhythmias	29 (6)
– Chronic atrial arrhythmias	48 (10)
– Other	5 (1)

Data are *n* (%) or median (range)

*BMI* body mass index

Of these 497 patients, 83 (17%) had an episode of chest pain at first presentation to the ED, whereas 63 (13%) presented with syncope or mentioned experiencing syncope before presentation to the ED (see Tables S1 and S2 in Electronic Supplementary Material). An electrocardiogram (ECG) was made in 464 patients (93%), of whom 29 (6%) presented with new-onset atrial arrhythmias (25 with atrial fibrillation and 4 with atrial flutter) and 5 (1%) with other new-onset arrhythmias (1 with ventricular fibrillation, 1 with ventricular tachycardia, 1 with asystole and 2 with supra-ventricular tachycardia) (see Table S3 in Electronic Supplementary Material).

### Chest pain

**Fig. 1:**
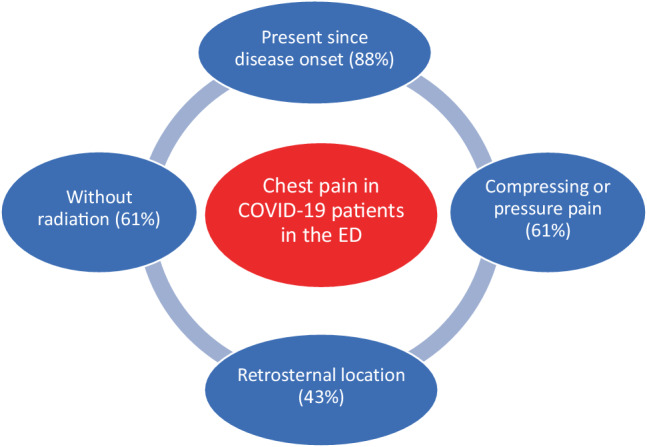
Main features of chest pain in COVID-19 patients in the emergency department (*ED*)

We found a significant difference in median age upon presentation between patients with chest pain (61 years) and those without chest pain (73 years) (*p* < 0.001). In 51 patients (61%), the amount of information recorded in the EHR was considered sufficient to study the chest pain characteristics. Of these 51 patients, 45 (88%) stated they had chest pain since the beginning of their illness, while 6 (12%) developed chest pain during their illness (Fig. 1). The 51 patients described their chest pain as compressing/pressure pain (*n* = 31; 61%), a sharp/stabbing pain (*n* = 8; 16%) or another type of pain, such as a cramping or burning pain (*n* = 12; 23%).

With regard to the location of the chest pain, 22 patients (43%) described this as retrosternal, 6 (12%) as diffuse around the chest area and 4 (8%) as unilateral; the location was unclear in the EHRs of the remaining 19 patients (Fig. 1). In 31 patients (61%), the pain did not radiate, whereas 11 (22%) felt the pain spread to their cheeks, arms or in between shoulders; this information was missing for 9 patients (18%).

According to 18 patients (35%), pain provoking factors were: heavy coughing (*n* = 5), palpation and deep inhalation (*n* = 4) and exercise (*n* = 5); 2 patients could not categorise their pain. No provoking factors were mentioned for the remaining 33 patients (65%).

### Syncope and arrhythmia

Patients with syncope were older (75 vs 72 years; *p* = 0.017), had a shorter duration of symptoms (5 vs 7 days; *p* < 0.001) and reported fewer respiratory complaints (68% vs 90%; *p* < 0.001) than those without syncope (see Table S2 in Electronic Supplementary Material).

Patients with new-onset atrial arrhythmias also presented with a shorter duration of symptoms (5 vs 7 days; *p* = 0.013) and fewer respiratory complaints (72% vs 89%; *p* = 0.012) than patients who presented without arrhythmias. In addition, patients with new-onset atrial arrhythmias more frequently had a history of cardiovascular disease (79% vs 50%; *p* = 0.003) (see Table S3 in Electronic Supplementary Material).

## Discussion

In this single-centre observational study, we aimed to assess cardiac symptoms of COVID-19 patients upon first presentation to the ED and found they can present with chest pain, syncope or new-onset arrhythmias. This chest pain could be best described as a non-radiating, compressing pain that was mainly located retrosternally and had been present since the beginning of the illness. Patients with chest pain were significantly younger than those who presented without chest pain.

Furthermore, patients who presented with syncope were older, had a shorter duration of symptoms and had fewer respiratory complaints than those without syncope. Similarly, patients with new-onset atrial arrhythmias presented with a shorter duration of symptoms and fewer respiratory complaints. As syncope and arrhythmias were present early in the disease course of COVID-19, it may be harder to recognise them as COVID-19 symptoms, especially in the absence of typical symptoms such as respiratory complaints.

At this moment, the follow-up of chest pain in COVID-19 patients should not differ from routine follow-up of patients presenting with chest pain to the ED (f.e. ECG and laboratory evaluation of cardiac markers). Based on a patient’s history, chest pain in COVID-19 cannot be differentiated from chest pain due to other causes (i.e. acute coronary syndrome, pulmonary embolism or pneumonia). Hence, in patients presenting to the ED with cardiac symptoms, clinicians should be wary of the possibility of an underlying SARS-CoV‑2 infection.

Our study makes way for further research on chest pain characteristics in COVID-19, which may find differences that can adapt routine follow-up in patients presenting to the ED with chest pain and recent positive COVID-19 test results.

Moreover, chest pain is reported by 22% of patients with long COVID (i.e. COVID symptoms persisting after three months) [21]. Cardiac injury following SARS-CoV‑2 infection (subclinical myocardial infarction followed by diastolic dysfunction) may be prevented if typical characteristics of chest pain in COVID-19 are clarified and appropriate cardiac care is started earlier [21, 22]. Long-time follow-up research is needed to improve recognition of cardiac manifestations of COVID-19 and cardiac care in COVID-19 patients.

### Study limitations

The first limitations of this study were that we mainly examined patients with severe COVID-19 who had been referred to the ED by a GP and that our hospital is located in a region that had a very high incidence of COVID-19. Hence, this study may have been subjected to selection bias, and our observations may therefore not apply to milder cases of COVID-19. In addition, the numbers of patients and events in this study were small, and the observational and single-centre design may have contributed to unobserved confounding bias.

A major limitation was the lack of additional diagnostic testing to assess thrombo-embolic events. At the time of data collection, research on these complications was lacking, and we did not routinely perform additional tests to rule out thrombo-embolic events as the cause of chest pain in our population. This could be a source of bias, as chest pain and syncope are symptoms of pulmonary embolism, which could have offered a sound explanation.

## Supplementary Information

**Table S1** Baseline characteristics of patients with chest pain

**Table S2** Baseline characteristics of patients with syncope

**Table S3** Baseline characteristics of patients with arrythmias
