# Supplementary material for: Characteristics of chest pain in COVID-19 patients in the emergency department
Source: Neth Heart J. 2022 Oct 21;30(11):526–32. doi: 10.1007/s12471-022-01730-7 (PMC9589604; doi:10.1007/s12471-022-01730-7)
Supplement: Supplementary file 1 — Table S1 Baseline characteristics of patients with chest pain [file 12471_2022_1730_MOESM1_ESM.docx]

**Table S1** Baseline characteristics of patients with chest pain

|  | Chest pain | No chest pain | p-value |
| --- | --- | --- | --- |
| **Baseline characteristics** |  |  |  |
| Male, no. (%) | 51 (61) | 266 (64) | P=0.619 |
| Age in years, median (range) | 61 (27-90) | 73 (31-94) | P<0.001* |
| Symptom duration in days, median (range) | 7 (0-22) | 7 (0-22) | P=0.485 |
| **Comorbidities** |  |  |  |
| Cardiovascular disease, no. (%) | 29 (35) | 227 (55) | P=0.001* |
| Pulmonary disease, no. (%) | 21 (25) | 108 (26) | P=1.000 |
| Renal disease, no. (%) | 7 (8) | 90 (22) | P=0.004* |
| Hypertension, no. (%) | 34 (41) | 225 (54) | P=0.030* |
| Diabetes mellitus, no. (%) | 11 (13) | 91 (22) | P=0.075 |
| Active malignancy, no. (%) | 2 (2) | 33 (8) | P=0.097 |
| Obesity, no. (%) | 33 (40) | 118 (29) | P=0.050 |
| **Symptoms** |  |  |  |
| Fever, no. (%) | 64 (77) | 335 (81) | P=0.450 |
| Respiratory complaints, no. (%) | 70 (84) | 365 (88) | P=0.362 |
| Gastrointestinal complaints, no. (%) | 42 (51) | 219 (53) | P=0.719 |
