# Supplementary material for: Characteristics of chest pain in COVID-19 patients in the emergency department
Source: Neth Heart J. 2022 Oct 21;30(11):526–32. doi: 10.1007/s12471-022-01730-7 (PMC9589604; doi:10.1007/s12471-022-01730-7)
Supplement: Supplementary file 2 — Table S2 Baseline characteristics of patients with syncope [file 12471_2022_1730_MOESM2_ESM.docx]

**Table S2** Baseline characteristics of patients with syncope

|  | Syncope | No syncope | p-value |
| --- | --- | --- | --- |
| **Baseline characteristics** |  |  |  |
| Male, no. (%) | 40 (63) | 277 (64) | P=1.000 |
| Age in years, median (range) | 75 (46-94) | 72 (27-94) | P=0.017* |
| Symptom duration in days, median (range) | 5 (0-22) | 7 (0-22) | P<0.001* |
| **Comorbidities** |  |  |  |
| Cardiovascular disease, no. (%) | 35 (56) | 221 (51) | P=0.504 |
| Pulmonary disease, no. (%) | 13 (21) | 116 (27) | P=0.358 |
| Renal disease, no. (%) | 13 (21) | 84 (19) | P=0.865 |
| Hypertension, no. (%) | 36 (57) | 223 (51) | P=0.420 |
| Diabetes mellitus, no. (%) | 16 (25) | 86 (20) | P=0.318 |
| Active malignancy, no. (%) | 3 (5) | 32 (7) | P=0.602 |
| Obesity, no. (%) | 15 (24) | 136 (31) | P=0.244 |
| **Symptoms** |  |  |  |
| Fever, no. (%) | 46 (73) | 353 (81) | P=0.129 |
| Respiratory complaints, no. (%) | 43 (68) | 392 (90) | P<0.001* |
| Gastrointestinal complaints, no. (%) | 30 (48) | 231 (53) | P=0.421 |
