# Supplementary material for: Characteristics of chest pain in COVID-19 patients in the emergency department
Source: Neth Heart J. 2022 Oct 21;30(11):526–32. doi: 10.1007/s12471-022-01730-7 (PMC9589604; doi:10.1007/s12471-022-01730-7)
Supplement: Supplementary file 3 — Table S3 Baseline characteristics of patients with arrythmias [file 12471_2022_1730_MOESM3_ESM.docx]

**Table S3** Baseline characteristics of patients with arrythmias

|  | New-onset Afib/Aflut | Other | p-value |
| --- | --- | --- | --- |
| **Baseline characteristics** |  |  |  |
| Male, no. (%) |  |  | P=0.691 |
| Age in years, median (range) | 76 (35-91) | 72 (27-94) | P=0.118 |
| Symptom duration in days, median (range) | 5 (1-14) | 7 (0-22) | P=0.013* |
| **Comorbidities** |  |  |  |
| Cardiovascular disease, no. (%) | 23 (79) | 218 (50) | P=0.003* |
| Pulmonary disease, no. (%) | 8 (28) | 114 (26) | P=0.830 |
| Renal disease, no. (%) | 7 (24) | 82 (19) | P=0.469 |
| Hypertension, no. (%) | 19 (66) | 222 (51) | P=0.178 |
| Diabetes mellitus, no. (%) | 9 (31) | 88 (20) | P=0.164 |
| Active malignancy, no. (%) | 3 (10) | 29 (7) | P=0.440 |
| Obesity, no. (%) | 10 (34) | 133 (31) | P=0.680 |
| **Symptoms** |  |  |  |
| Fever, no. (%) | 21 (72) | 349 (80) | P=0.339 |
| Respiratory complaints, no. (%) | 21 (72) | 389 (89) | P=0.012* |
| Gastrointestinal complaints, no. (%) | 10 (34) | 232 (53) | P=0.056 |
